# Sense of support within the family: a cross-sectional study of family members in palliative home care

**DOI:** 10.1186/s12904-020-00623-z

**Published:** 2020-08-07

**Authors:** Anna Milberg, Maria Liljeroos, Rakel Wåhlberg, Barbro Krevers

**Affiliations:** 1 Department of Health, Medicine and Caring Sciences, Linköping University, Linköping, Sweden; 2 Department of Advanced Home Care, Linköping University, Norrköping, Sweden; 3 Centre for Clinical Research Sörmland, Uppsala University, Eskilstuna, Sweden; 4 Medical department, Mälarsjukhuset hospital, 631 88 Eskilstuna, Sweden

**Keywords:** Palliative home care, Family members, Support, Family system theory

## Abstract

**Background:**

Despite evidence that family members’ support to each other can be of importance to its members, there are limited studies of factors related to family members’ sense of such support during palliative care.

**Aim:**

Based on the family systems approach, we evaluated which factors were associated with family members’ sense of support within their closest family in a palliative home care context and developed a model that predicts such sense of support.

**Design:**

A cross-sectional design was used. We interviewed 209 adult family members (69% of eligible) of adult patients with expected short survival receiving palliative home care.

**Methods:**

Generalised linear models were used to evaluate individual factors related to family members’ sense of support within their closest family during palliative care. The Akaike Information Criterion (AIC) was applied in the model-building analyses.

**Results:**

Nineteen variables were identified that were significantly associated with the family members’ sense of support within the closest family. Model building selected six variables for predicting this sense of support (decreasing Wald values): family member perceiving support from other more distant family members; feeling secure with the provided palliative home care; possibility of respite if family member needed a break; family member living alone; being a child of the patient (inverse relationship); perceiving that the patient was supported by other family members.

**Conclusions:**

Our findings support clinical application of the Family Systems Theory in the context of palliative care. The factors identified may be of value in assisting practitioners in detecting and treating family members sensing a low level of support within the closest family.

## Background

When an ill individual is approaching death, the situation often constitutes a crisis for the whole family [1–5], and an increased risk for psychological morbidity in family members [6, 7]. When confronted with such life events, family members’ support to each other are of great importance to the individual members of the family as well as to the family’s proper functioning. Previous research has demonstrated that families experiencing high level of support and cohesion present lower levels of psychosocial morbidity and more effective social adjustment than families experiencing lower levels of support, both during palliative care and in bereavement [8–10].

There is growing evidence evolving in palliative care research that family members and patients constitute an interdependent relational system [4, 11, 12], which stresses the significance of family members perceiving a sense of support from those closest to them [8–10, 13]. There is also an advance in theories, such as Family Systems Theory, which emphasise the significance of such support within a family, since significant change or event in one family member affects all other family members [2, 3, 14].

In palliative home care, family members play an integral role in symptom assessment, monitoring, and delivery of complex therapeutic interventions. Further, death and dying should be perceived as a family concern that throws the family out of balance and requires adjustment of all family members to the new family reality [2, 15]. To ensure a link between family research and practice, a conceptual framework is vital in order to comprehend observations made, select proper supportive family interventions, and to evaluate their effectiveness [2]. Also, by using a framework, the identification of individuals at risk of inadequate support within the family may improve.

### The family systems theory

Family Systems Theory focuses primarily on the interaction between members of the family and between the family and other systems. A system can be defined as a set of interacting elements. The family is seen as a group of interdependent individuals, making the family a system, and a change in one family member, e.g. when someone in the family becomes critically ill, will influence the whole system [15].

A central concept in Family Systems Theory is that the family is greater than the sum of its parts. A family system is part of a larger suprasystem and it is itself further composed of many subsystems [15]. The family cannot be fully understood by the examination of individual members or subsystems in isolation from each other and from the suprasystems of which the family forms a part. In palliative care, communities, the healthcare system and the palliative home care unit are examples of suprasystems within which the family is nested. It is important to understand how these parts relates to understand the family and its needs as a whole.

Another important concept in Family Systems Theory is that the family can create a balance between change and stability. Families in palliative care often live with uncertainties related to symptom management and the patient’s life expectancy. This may place them in a constant state of anxiety and unbalance, and they often struggle with finding such balance [2].

Hence, there is clear evidence for the significance of family members’ perceptions of support from those closest to them during palliative care, and support for the relevance of a systemic perspective such as Family System Theory for enhancing palliative care practice. However, there are few theory-based studies with focus on factors that are related to family members’ sense of such support in a palliative home care context. Previous research has mostly focused on the relation between the informal caregiver and the patients, and in the in-hospital palliative care context [2, 4]. Since there is an international move from in-hospital palliative care towards palliative home care to meet patient and family choice, it is important to increase the knowledge on how to best support families in such context.

Therefore, our aim was, using the family systems approach, to study which factors are associated with family members’ sense of support within the closest family in the palliative home care context, and to develop a model that predicts their sense of such support.

### Main hypothesis

With the intention of testing our hypotheses we formulated three assumptions based on The Family Systems Theory:

1. the family members’ sense of support within their closest family was a marker of the *subsystem Family member - Closest family*, i.e. the relationship between the family member and other members of the family whom he or she was closest to emotionally, and that this subsystem was valuable for the functioning of the family;
2. there was a dynamic relationship between the different elements of the family system, and a change in one element of the *subsystem Family member - Closest family* (i.e. *Family member characteristics* and *Patient characteristics*) would influence other elements within this subsystem;
3. a change in the *subsystem Family member - Closest family* would influence other subsystems (i.e. *subsystem Family member - Other more distant family members* and *subsystem Patient-Other family members* (than the family member interviewed))*,* and *suprasystems* (i.e. in this study *suprasystem Family member - Palliative home care unit*)*.*

**Fig. 1:**
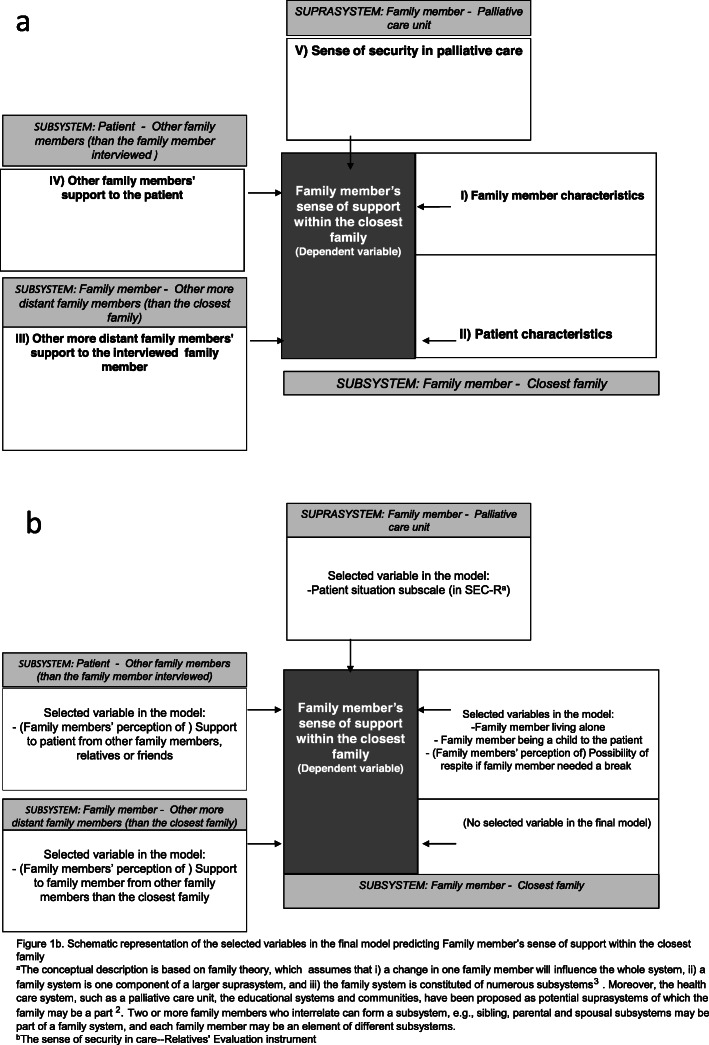
**a-b.** A theoretical conceptual description of the hypothesised relationships formulated before the study (Fig. 1a) and a schematic representation of the selected variables in the final model predicting family member’s sense of support within the closest family (Fig. 1b). It was hypothesised that the family member’s sense of support within the closest family (dependent variable; dark grey box) would be associated with five domains (in white boxes), each domain representing a subsystem or suprasystem (in light grey boxes)

We hypothesised that the family members’ sense of support within the closest family, in a population of family members of patients with short expected survival (the coming months), would be associated with the following five domains, each domain representing a subsystem or suprasystem: *“Family member characteristics; “Patient characteristics”, “Other more distant family members’ support of the interviewed family member”; “Other family members’ support of the patient”;* Family member’s sense of *“security in palliative care.* A theoretical conceptual description of the hypothesised relationships was formulated by the authors before the study (Fig. 1a).

Our hypotheses were grounded in previous research that proposed that family members’ sense of support within the family, when the patient has advanced cancer or been offered palliative care, is related to gender, socioeconomic factors, cultural diversity [16], psychosocial morbidity [8–10], and quality of life [16]. Moreover, we hypothesised that age [10, 17], changed behaviour [18], coping [13], self-efficacy [12, 19], concern about personal finances [17], perceived quality of the palliative care received [20], and attachment security [21, 22] would be related to the family members’ sense of support within the closest family as these factors have been described as significant in palliative care, in cancer or dementia care or in relation to the patients’ sense of support within the closest family in a palliative care context [23].

## Methods

### Study population and procedures

In this cross-sectional study, the participants consisted of family members of patients currently receiving palliative home care. Details of the data collection have already been published and is briefly summarised here [20, 24, 25]. The participants were recruited from three specialised palliative home care units and three primary care-based palliative home care units in Sweden. Three of the units were advanced palliative home care teams (with a multiprofessional team that included 24-h services and access to a backup ward) and three were primary-care-based teams with a palliative care consultant and a specialist nurse available during the daytime. The staff of the palliative care team assessed the inclusion and exclusion criteria for all of the patients who were admitted to the participating palliative units. The eligible participants received written study information and were asked by a staff member of the palliative care team if they wanted to participate. The five interviewers were staff experienced in palliative care but not involved in the participants medical care.

**Fig. 2:**
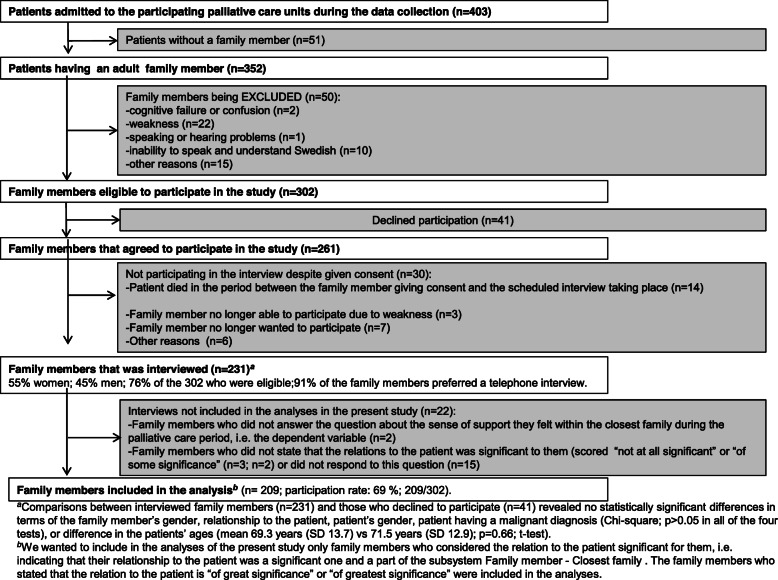
Overview of study enrolment

The analyses were based on a sample of 209 participants (69% of the 302 eligible family members; 209/302). See overview of study enrolment in Fig. 2. The demographic characteristics of these 209 family members and patients are displayed in Tables 1 and 2. The data were collected between September 2009 and October 2010 and has resulted in several previous papers [20, 24, 25]. Due to limited research time (teaching and clinical work), this manuscript turned out slow in the making.

**Table 1:** Variable domain 1. Family characteristics. Sample characteristics of the interviewed family members (*n* = 209) and analysis of the individual variables in Domain 1. Family characteristics (subsystem Family member -Closest family) in relation to the family members’ sense of support within the closest family (dependent variable)

Variable domain *(relating subsystem or suprasystem)*	Variable (range of response alternatives/index)	% responses	Description (mean [SD];range) or percent)	Wald values^***a***^ for individual variable	Relation-ship
**I. Family member characteristics** *(SUBSYSTEM Family member-Closest family)*	*Demographics:* Age (in years)	99	60.5 [13.3];(21–87)	2.34	
	Gender: male/female	99	45%/55%	0.02	
	Married or partner/ single	100	89%/ 11%	0.13	
	Living conditions				
	Alone	99	11%	*5.94*	pos
	with husband, wife or cohabitant	99	84%	2.71	
	with children	99	12%	*4.53*	neg
	with siblings or others	99	3%	0.29	
	Have children	100	88%	1.19	
	Native born in Sweden	100	92%	1.68	
	Education (highest level completed) (1–6)	100		0.11	
	1(No formal)/ 2(Basic education only)/		3%/ 21%/		
	3(High school)/ 4(Vocational education/		22%/ 20%/		
	5(University< 3 years)/ 6(University 3 years or more)		9%/ 23%		
	Relation to the patient				
	Husband, wife or partner		64%	0.92	
	Child		31%	3.26	
	Other^*b*^		6%		
	Main occupation				
	Employed	100	50%	2.57	
	Caring for family member with reimbursement	100	10%	0.48	
	Caring for family member without reimbursement	100	7%	0.43	
	Age pensioner	100	40%	*6.86*	pos
	*Health-related quality of life*				
	EQ 5D index (−0.594 (worst possible) – 1.00 (best possible))	100	0.74[0.23];(−0.02–1)	1.98	
	Mobility (1 (no problems - 3 (severe problems))	100	1.16[0.38];(1–3)	0.19	
	Self-care (1 (no problems - 3 (severe problems))	100	1.02[0.14];(1–2)	2.60	
	Usual activities (1 (no problems - 3 (severe problems))	100	1.10[0.32];(1–3)	0.02	
	Pain/Discomfort (1 (no problems - 3 (severe problems))	100	1.68[0.59];(1–3)	0.40	
	Anxiety/Depression (1 (no problems - 3 (severe problems))	100	1.68[0.58];(1–3)	*7.08*	neg
	General health (from SF-36; 1(excellent) - 5 (bad))	100	2.79[1.11];(1–5)	2.67	
	General Quality of life (from WHO QOL 100; 1(very bad) - 5 (very good))	100	3.66[0.90];(1–5)	*5.90*	pos
	*Situation as family member to a severely ill person*				
	Type of support the family member provided to the ill person				
	Health care	100	63%	1.89	
	Physical, personal care	99	48%	0.67	
	Transport	100	74%	0.87	
	Emotional, social support	100	98%	0.64	
	Support in home and household	100	80%	0.27	
	Support with financial management	100	59%	0.53	
	Financial support	100	19%	0.23	
	Organise care and support	100	69%	0.00	
	Extent of support the family member provided to the ill person (1–6)	98		0.49	
	1(Around-the-clock)/ 2(Always daytime)/ 3(Several times per day		32%/ 10%/ 20%		
	4(Once per day)/ 5(Some time per week)/ 6(No need)		11%/ 7%/ 5%/ 13%		
	Family members’ perception of being a family caregiver				
	Negative impact (COPE^*c*^_NEG) (4–28)	100	10.48[3.02];(7–22)	*12.50*	neg
	Positive value (COPE_POS) (4–16)	99	14.08[1.81];(6–16)	*12.52*	pos
	Quality of support (COPE_Support) (4–16)	99	12.58[2.60];(5–16)	*22.12*	pos
	Possibility of respite if family member needed a break (1–3)	100			
	(1(no)/ 2(yes, with some difficulty)/ 3(yes, easy))		15%/ 43%/ 42%/	*16.39*	pos
	Possibility of respite if family member turned ill (1–3)	100			
	(1(no)/ 2(yes, with some difficulty)/ 3(yes, easy))		21%/ 37%/ 42%	*6.47*	pos
	*Attachment security:*				
	Anxiety dimension (ECR-M16^*c*^; 1–7)	99	2.81[1.05];(1–6.25)	*6.27*	neg
	Avoidance dimension (ECR-M16; 1–7)	98	2.82[1.11];(1–6.25)	2.59	
	*Stress and coping*				
	Nervousness and stress (from PSS^*c*^; 1 (never) - 5 (very often))	100	3.0[1.18];(1–5)	*6.71*	neg
	Too many problems to manage (from PSS; 1 (never) - 5 (very often))	100	2.19[1.11];(1–5)	2.74	
	Worry about private economy (1 (never) - 5 (very often))	100	1.70[1.11];(1–5)	0.23	
	Self-efficacy (from GSE^*c*^; 1 (fully disagree) - 4 (fully agree))	100	3.28[0.67];(1–4)	2,37	
	Religious or existential faith that helps (1 (fully disagree) - 4 (fully agree))	100	2.26[1.08];(1–4)	1.83	

^a^Wald =4 is approximately equivalent with *p* = 0.05; Wald = 6 is approximately equivalent with *p* = 0.01

^b^Too diverse in data (merged categories) to be meaningful to compute

^c^SEC-R, The sense of security in care-Relatives’ Evaluation instrument; COPE, assessment of negative impact, positive value and quality of support of caregiving in informal carers of older people; ECR-M16, Experiences in Close Relationships scale; EQ-5D, EuroQol-5D; PSS, Perceived Stress Scale; GSE, General Self-Efficacy Scale

**Table 2:** Variable domains 2–5. Sample characteristics and analysis of the individual variables in Domain 2–5 in relation to the family members’ sense of support within the closest family (dependent variable) Sample characteristics of patients (of the interviewed family members; *n* = 209) and analysis of the individual variables in relation to the family members’ sense of support within the closest family (dependent variable)

Variable domain (*relating subsystem or suprasystem*)	Variable (range of response alternatives/index)	% responses	Description (mean [SD]; range) or percent	Wald values^**a**^ for individual variable	Relation-ship
**II. Patient characteristics** (*SUBSYSTEM: Family member -Closest family)*	*Patient demographics*				
	Patient’s age (in years)	94	68.5 [13.77]; (20–94)	0.53	
	Patient’s gender: male/ female	94	39%/ 55%	1.29	
	Patient native born in Sweden	99	88%	0.30	
	Patient’s living conditions				
	Alone	98	27%	2.07	
	With wife/husband/co-habitant	98	65%	0.04	
	With child	98	5%	*4.40*	neg
	With other	98	8%	3.44	
	Geographical distance between housing of patient and family member	99		0.44	
	Same household/ 5–60 min distance/More than 1 h		62%/ 30%/ 6%		
	*Patient illness*				
	Patient having difficulties with memory (1 (never) – 5 (very often)	99	2.16 [1.27]; (1–5)	0.48	
	Patient having changed behaviour (1 (never) - 5 (very often))	99	2.39 [1.18];(1–5)	*5.09*	neg
	Malignant diagnoses				
	Gastro-intestinal	94	30	0.02	
	Respiratory	94	11%	2.85	
	Breast	94	11%	0.29	
	Gynaecological	94	6%	0.21	
	Urological	94	13%	0.19	
	Haematological	94	6%	1.69	
	Other malignancies	94	10%	0.72	
	Non-malignant diagnoses^*b*^	94	7%	0.18	
	Time since diagnosis (in months)	99	35.0 [44.89];1–240)	0.03	
**III. Other more distant family members’ support to the interviewed family member** *(SUBSYSTEM: Family member - Other more distant family member) s*	Support to family member from other family members than the closest family (1 (never) - 6 (always))	98	4.68 [1.16];(2–6)	*50.24*	pos
**IV. Other family members’ support to patient** *(SUBSYSTEM: Patient – Other family members (than the interviewed family member)*	Support to patient from other family members, relatives or friends (1 (never) - 6 (always))	97	4.57 [1.25];(1–6)	*43.97*	pos
**V. Sense of security in palliative home care** *(SUPRASYSTEM: Family member - Palliative home care unit)*	*The family members’ sense of security with palliative care:* Care interaction subscale (SEC-R^*c*^; 1 (never) - 6 (always))	99	5.12 [0.70];(1–6)	*4.55*	pos
	Mastery subscale (SEC-R; 1 (never) - 6 (always))	100	4.20 [0.96];(1.6)	*8.42*	pos
	Patient situation subscale (SEC-R; 1 (never) - 6 (always))	100	4.82 [0.76];(1–6)	*21.17*	pos
	Time from commencement of palliative home care to the interview with the family member (days)	93	187 [377.7];(11–4256)	1.72	

^a^Wald =4 is approximately equivalent with *p* = 0.05; Wald = 6 is approximately equivalent with *p* = 0.01

^b^Neurological disease (*n* = 9), heart- or lung disease (*n* = 4), and other (*n* = 2)

^c^SEC-R, The sense of security in care-Relatives’ Evaluation instrument; ECR-M16, Experiences in Close Relationships scale

### Measures

#### Dependent variable

The family members’ sense of support within the closest family in the palliative care context (dependent variable), as a marker of the *subsystem Family member - Closest family*), was assessed by one question constructed by the authors (6-point response scale; 1 (never) – 6 (always). Our intention was to prevent exclusion of single individuals not having a conventional nuclear family; therefore, we used a wide definition of the family [2] and let the family members themselves select which subsystem was the most significant, by avoiding the word ‘family’ in the question: “How often do you and those closest to you give support to each other?”

#### Independent variables in five domains

The five domains possibly related to the dependent variable were evaluated by the variables listed in Table 3.

**Table 3:** Overview of measures. The five domains potentially related to the dependent variable (The family members’ sense of support within the closest family) were evaluated by the variables listed in the table

Variable domain (*relating subsystem or suprasystem)*	Measures	Origin
**I. Family member characteristics** *(SUBSYSTEM* *Family member - Closest family)*	*Demographics*: age, gender, living and family conditions, education, country of birth, relationship to the patient, and main occupation	a
	*Health-related quality of life*: The EuroQol-5D (EQ-5D), including: mobility, self-care, pain, usual activities, psychological status; 3-point response scale: 1 (no problems) - 2 (some problems) - 3 (severe problems). Index score was calculated for each respondent’s health status: 1 = full health; − 0.594 = worst-imaginable health state	1
	General quality of life: the WHO QOL 100: one question; 5-point scale: 1 (very poor) – 5 (very good)	2
	General health: one overall question from the SF-36; 5-point scale: 1 (excellent health) – 5 (poor health)	3
	*Situation as family member to a severely ill person:*	
	Type of support/care the family member provided to the ill person: eight alternatives; yes/no (see Table 1)	a
	Extent of support the family member provided to the ill person: one question; 6-point scale: 1 (around-the-clock) – 6 (no need of support)	a
	The family member’s perception of being a family caregiver: the COPE questionnaire: 15 questions; 4 point scale: 1 (never) – 4 (always) based on 3 validated sub-scales: Negative impact scale, Positive value scale and Quality of support scale	4
	Possibility of respite if family member needed a break: one question; 3-point scale: 1 (no) – 2 (yes, with some difficulty) – 3 (yes, easy)	a
	Possibility of respite if family member became ill: one question; 3-point scale: 1 (no) – 2 (yes, with some difficulty) – 3 (yes, easy)	a
	*Attachment security*: The Experiences in Close Relationships scale (ECR-M16); 16 items to measure attachment anxiety (fear of rejection and abandonment); mean value of 8 items and avoidance (discomfort with closeness and dependence on close others; mean value of 8 items) in close relationships (including non-romantic partners); 7-point scale: 1 (lower attachment insecurity) - 7 (greater attachment insecurity)	5
	*Stress and coping:* Stress: two (of ten) items from the Perceived Stress Scale (PSS); 5-point scale: 0 (never) – 4 (very often)	6
	Worry about personal finances during the last month: 5-point scale: 0 (never) – 4 (very often)	a
	Self-efficacy: One statement (of ten) from the General Self-Efficacy Scale (GSE); 4-point scale: 1 (not at all true) – 4 (exactly true)	7,8
	Religious or existential belief that helps the informant to cope with problems: One statement; 4-point scale: 1 (not at all true) – 4 (exactly true)	a
**II. Patient characteristics** (*SUBSYSTEM: Family member - Closest family)*	*Patient demographics*: age, gender, living and family conditions, country of birth, geographical distance to family member	a
	*Patient illness:* Patient diagnosis and the time since diagnosis.	b
	Patient having difficulties with memory (according to the family member’s perception): one question;5-point response scale: 1 (never) – 5 (very often)	a
	Patient having changed behaviour: one question; 5-point response scale (according to the family member’s perception): 1 (never) – 5 (very often)	a
**III. Other more distant family members’ support to the interviewed family member** *(SUBSYSTEM: Family member - Other more distant family members (than the closest family))*	The family member’s perception of support from members of the family, relatives and friends other than those closest to them: one question; 6-point response scale: 1 (never) – 6 (always)	a
**IV. Other family members’ support to the patient** *(SUBSYSTEM: Patient - Other family members (than the interviewed family member)*	The family members’ perception of the patient being supported by family members other than those who were interviewed: one question; 6-point response scale: 1 (never) - (always)	a
**V. Sense of security in palliative home care** *(SUBSYSTEM: Family member – Palliative home care unit)*	*The family members’ sense of security with palliative care:* The sense of security in care-Relatives’ Evaluation instrument (SEC-R;15-item instrument (6-point scale: 1 (never) - 6 (always) based on 3 validated sub-scales: Care Interaction (eight items), Mastery (four items) and Patient Situation (three items)	9
	Time (days) from commencement of palliative home care services to the interview (with the family member)	b

^a^Developed by the authors, ^b^Medical record

1. Brooks, R., *EuroQol: the current state of play.* Health Policy, 1996. **37**(1): p. 53–72.

2. TheWHOQOLgroup, *The World Health Organization Quality of Life Assessment (WHOQOL) Development and general psychometric properties.* Soc Sci Med, 1998. **46**: p. 1569–1585.

3. Brazier, J.E., et al., *Validating the SF-36 health survey questionnaire: new outcome measure for primary care.* Bmj, 1992. **305**(6846): p. 160–4.

4. Balducci, C., et al., *Negative impact and positive value in caregiving: validation of the COPE Index in a six-country sample of carers.* Gerontologist, 2008. **48**: p. 278–286.

5. Lo, C., et al., *Measuring attachment security in patients with advanced cancer: psychometric properties of a modified and brief Experiences in Close Relationships scale.* Psychooncology, 2009. **18**: p. 490–499.

6. Cohen, S., T. Kamarck, and R. Mermelstein, *A global measure of perceived stress.* J Health Soc Behav, 1983. **24**(4): p. 385–96.

7. Bosscher, R.J. and J.H. Smit, *Confirmatory factor analysis of the General Self-Efficacy Scale.* Behav Res Ther, 1998. **36**(3): p. 339–43.

8. Love, J., C.D. Moore, and G. Hensing, *Validation of the Swedish translation of the General Self-Efficacy scale.* Qual Life Res, 2012. **21**(7): p. 1249–53.

9. Krevers, B. and A. Milberg, *The sense of security in care--Relatives’ Evaluation instrument: its development and presentation.* J Pain Symptom Manage, 2015. **49**(3): p. 586–94

To be mindful of the family members’ situations in having a close family member (i.e. the patient) who was dying, we used short scales or single questions where feasible. Most of the data was gathered by a structured interview with the family member using questionnaires that were administered verbally, and some data were collected via the palliative care team, for example the patients’ diagnoses.

### Statistical analyses

We conducted analytical tests to compare the family members who were interviewed with those who declined to participate (chi-square and t-test).

The percentage of missing values ranged from 0% (e.g. on the dependent variable) to 6.7% (14/209; Time from admittance to palliative care unit). If there were missing values, the specific analysis was conducted without this participant’s value, though the participant could be included in other analyses. The respondents scarcely used a few of the response options, (e.g., only one participant responded “never” on the dependent variable). Such response options were not relevant to include in the analyses and were fused with the next response option.

We calculated descriptive statistics for individual variables in the five domains. Thereafter we assessed the relationship between the dependent variable and the independent variables in the five domains by Wald values from generalised linear models (ordinal multinomial distribution and logit link). The Akaike Information Criterion (AIC) was applied in the model-building analyses [26]. The AIC offers a relative measure of information lost when a given model is employed to portray reality.

One analysis per domain was carried out, as the first step in the model-building process, and we used AIC to determine in which sequence the domains should be considered. We allowed only variables with Wald values > 2, corresponding to approximately *p* < 0.15, to be included in the further model-building, i.e. a generous selection criterion to decrease the risk of rejecting variables that in further model-building could have been valuable. We took the domain with the lowest AIC first in the model-building analyses and the domain with the highest AIC last, because a lower AIC value signals a higher value of explanation. In the final step in the process we used the best subset analyses with AIC. We also calculated classification of the developed model and computed the percentage correct classifications of the observed cases. Data analyses were done using Statistica, Version 10 (Statsoft Inc., USA).

## Results

The family members and patients’ characteristics are displayed in Tables 1 and 2. The family members scores of their sense of support within the closest family had a mean value of 4.8 (standard deviation (SD) 1.1; range 1 (never) to 6 (always)).

### Variables related to dependent variable

According to the analyses of the individual variables, all the hypothesised domains were significantly related to the family members’ sense of support within the closest family (dependent variable) (Tables 1 and 2).

Twelve variables were positively related to the dependent variable (presented in order of decreasing Wald values; 50.24–4.55): support to the family member from other family members (than the closest family); support to the patient from family members, relatives or friends (other than the person interviewed); perception of quality of support as a family caregiver; Patient Situation subscale (SEC-R); possibility of respite if family member needed a break; positive value of being a family caregiver; Mastery subscale (SEC-R); being an old-age pensioner; possibility of respite if family member became ill; family member living alone; quality of life; Care Interaction subscale (SEC-R) (Table 1 and Table 2)*.*

Seven variables negatively related to the dependent variable (presented in order of decreasing Wald values; 12.50–4.40): negative perception of the impact of being a family caregiver; anxiety/depression subscale; family member being nervous and feeling stressed; attachment anxiety; patient having changed behaviour; family member living with child/children; and patient living with child/children (Table 1 and Table 2).

### Model building

We began the model building with one analysis per domain, except for the *Family member characteristics* and the *Patient characteristics* domains. The variables in these two domains were thought to be too many, compared with the number of respondents, to be computed at the same time. Therefore, in Step 0, we split the variables in these domains into subdomains, calculating Wald values for each subdomain, and were chose only the variables with Wald > 2 for further analyses in Step 1, Table 4.

**Table 4:** Step-wise analyses (Best subset) for prediction of family member’s sense of support within the family (dependent variable) during the palliative home care period. Only variables in the five domains (each domain representing a subsystem or suprasystem) with Wald > 2 (see Table 1 and 2) were allowed to be included in the step-wise analyses

Variable domain *(relating subsystem or suprasystem)*	AIC	Variable	Wald value^***a***^ for partial regression coefficients							
			STEP 0	STEP 1	STEP 2	STEP 3	STEP 4	STEP 5	Step 6	STEP 7 Best subset
**IV. Other family members’ support to patient** *(SUBSYSTEM: Patient – Other family members (than the interviewed family member)*	506.1	Support to patient from other family members. Relatives or friends (1 (never) - 6 (always))		43.97	33.71	6.30	4.58	2.74	3.24	3.30^f^
**V. Sense of security in palliative home care** *(SUPRASYSTEM: Family member - Palliative home care unit)*	518.7	Care interaction subscale (SEC-R^*d*^; 1 (never) - 6 (always))		***1.12***						
		Mastery subscale (SEC-R; 1 (never) - 6 (always))		***0.14***						
		Patient situation subscale (SEC-R; 1 (never) - 6 (always))		14.22	11.82	9.71	9.94	8.30	10.02	9.98^f^
		Time from admittance to palliative care unit (days)		***0.28***						
**III. Other more distant family members’ support to the interviewed family member** *(SUBSYSTEM: Family member - Other more distant family member) s*	520.7	Support to family member from other family members than the closest family (1 (never) - 6 (always))		50.24		10.06	9.48	8.72	10.06	11.71^f^
**II. Patient characteristics**^**b**^ (*SUBSYSTEM: Family member -Closest family)*	523.9	*Demographics*								
	525.8	Patient’s age (in years)	***0.99***^***e***^							
		Patient’s gender: male/female	***1.19***							
		Patient native born i n Sweden (%)	***0.11***							
		Patient’s living conditions								
		Alone	***1.71***							
		With wife/husband/co-habitant	***0.48***							
		With child	5.04	3.44			***1.83***			
		With other	***0.04***							
		Geographical distance between housing of patient and family member								
		(Same household (1)-More than 1 h distance (6))	***0.04***							
		*Patient illness*								
	553.9	Malignant diagnoses	***0.44***							
		Respiration	2.39	***1.33***						
		Breast	***0.05***							
		Gynaecological	***0.63***							
		Urological	***0.64***							
		Haematological	***0.26***							
		Other malignancies	***0.12***							
	546.9	Non-malignant diagnoses^c^	***0.18***							
	576.0	Patient having difficulties with memory (1 (never) - 5 (very often))	***0.07***							
		Patient having changed behaviour (1 (never) - 5 (very often))	4.41	2.42			***0.001***			
		Time since diagnosis (in months)	***0.02***							
**I. Family member characteristics** *(SUBSYSTEM Family member-Closest family)*	534.4									
	578.7	*Demographics*								
		Age (in years)	3.11	3.33				2.59	***1.23***	
		Gender: male/female	***0.0003***							
		Married or partner/ single	5.55	2.52				***0.13***		
		Living conditions								
		Alone	2.61	2.74				5.07	8.13	7.29^f^
		with husband. Wife or cohabitant	2.48	***1.86***						
		with children	2.32	2.26				***1.50***		
		Have children	2.41	4.38				***1.00***		
		Native born in Sweden	***0.31***							
		Education (highest level completed) (No formal education (1)-University 3 yeats or more (6))	***0.24***							
		Main occupation								
		Employed	***0.04***							
		Self-employed	***1.07***							
		Caring for family member with grant	***0.30***							
		Caring for family member without grant	***0.32***							
		Old-age pensioner	4.95	2.17				2.01	***1.91***	
		Relation to the patient								
		Husband. wife or partner	***1.97***							
		Child	3.09	4.91				2.38	3.26	3.87^f.g^
	595.3	*Situation as family member to a severly ill person*								
		Type of support/care the family member provided to the ill person								
		Health care	***0.44***							
		Physical. personal care	***1.51***							
		Transport	***0.18***							
		Emotional. social support	***0.003***							
		Support in home and household	2.34	***0.50***						
		Support with financial management	***0.36***							
		Financial support	***0.02***							
		Organise care and support	***0.53***							
		Extent of support/attendance/care the family member provided to the ill person								
		(Around the clock (1) - No need (6))	***0.07***							
		Family members’ perception of being a family caregiver								
		Negative impact (COPE_NEG) (4–28)	2.07	***0.05***						
		Positive value (COPE_POS) (4–16)	***0.91***							
		Quality of support (COPE_SUPPORT) (4–16)	5.59	5.46				***0.13***		
		Possibility of respite if family member needed a break (Easy (1)- No (3))	5.37	10.66				6.61	6.40	7.67^f^
		Possibility of respite if family member turned ill (Easy (1)- No (3))	***0.25***							
	618.9	*Attachment security*								
		Anxiety dimension (ECR-M16^*d*^; 1–7)	5.24	2.15				***0.07***		
		Avoidance dimension (ECR-M16; 1–7)	2.01	***0.34***						
	638.6	*Health-related quality of life*								
		EQ 5D index^*d*^ (−0.594 (worst possible) – 1.00 (best possible))	***0.21***							
		General health (from SF-36; 1(excellent) - 5 (bad))	***0.19***							
		Quality of life (from WHO QOL 100; 1(very bad) - 5 (very good))	2.34	***1.19***						
	638.8	*Stress and coping*								
		Nervousness and stress (from PSS^*d*^; 1 (never) - 5 (very often))	4.22	3.54				2.03	***1.12***	
		Too many problems to manage (from PSS; 1 (never) - 5 (very often))	***0.05***							
		Worry about private economy (1 (never) - 5 (very often))	***0.10***							
		Self-efficacy (from GSE^*d*^; 1 (fully disagree) - 4 (fully agree))	***0.40***							
		Religious or existential faith that helps (1 (fully disagree) - 4 (fully agree))	***1.58***							

^a^Wald = 4 is approximately equivalent with *p* = 0.05; Wald = 6 is approximately equivalent with *p* = 0.01. Only variables with Wald> 2 were selected for further analyses

^b^The variables in both the “Characteristics of the patient” and the “Characteristics of the family member” domain were considered too many compared with number of respondents to be computed at the same time, so in Step 0 variables in these domains were divided into subdomains and Wald values for each subdomain were computed

^c^Neurological disease (n = 9). heart- or lung disease (*n* = 4). and other (*n* = 2)

^d^SEC-R. The sense of security in care-Relatives’ Evaluation instrument; COPE. assessment of negative impact. Positive value and quality of support of caregiving in informal carers of older people; ECR-M16. Experiences in Close Relationships scale; EQ-5D. EuroQol-5D; PSS. Perceived Stress Scale; GSE. General Self-Efficacy Scale

^e^Bold italic indicates variables that were excluded from the following steps

^f^Variable that was selected in the Best subset analyses

^g^Negative relationship to end-point

The AIC for the five domains resulted in “Other family members’ support to the patient” (Domain IV) was entered first in the stepwise procedure, and “Family member characteristics” (Domain I) last. The stepwise model building process resulted in a model with the following six variables (presented in order of decreasing Wald values): i) Support to family members from other family members than the closest family, Wald value 11.71 (i.e. a marker of the *subsystem Family member - Other more distant family members (than the closest family);* Domain III); ii) Family member feeling secure with the palliative home care, Wald value 9.98, (i.e. a marker of the *suprasystem Family member – Palliative care unit;* Domain V*)*; iii) Possibility of respite if family member needed a break, Wald value 7.67; iv) Family member living alone, Wald value 7.29; v) Family member being a child of the patient, Wald value 3.87 (; inversely related to dependent variable; iii-v being markers of the *subsystem Family member - Closest family*; Domain I)); vi) Support to patient from other family members, relatives or friends, Wald value 3.30 (i.e. as a marker of the *subsystem Patient - Other family members* (than the interviewed family member); Domain IV*)*. A schematic representation of the selected variables in the final model predicting Family member’s sense of support within the closest family is presented in Fig. 1b.

Of the 188 family members contributing to the final model, the correct response alternatives were predicted to 55% (range: 0% (response alternative 3 (sometimes) as 3 (sometimes) in 0 of 7) - 74% (6 (always) as 6 (always) in 51 of 69). Correct response plus minus one response alternative (e.g., response alternative 5 (very often) as either 4 (often), 5 (very often) or 6 (always)) was predicted in 92%. We thought it was valuable to prevent the risk of overrating a family member’s sense of support within his or her closest family; therefore, correct response minus 1 response alternative (e.g., response alternative 5 (very often) as either 4 (often) or 5 (very often) was calculated: 72%.

## Discussion

In this study, conducted in a palliative home care context, we have identified 19 variables that were associated with the family members’ sense of support within the closest family, and the final generalised linear model consisted of six variables for predicting this sense of support. The Family Systems Theory seems to fit the identified factors that affected family members’ sense of support within the closest family, and our findings support the clinical relevance of applying Family Systems Theory during palliative care.

The findings indicated that many family members of dying patients cared for by palliative home care units frequently sense support within their families, and that those family members who do not sense such support may feel stress, anxiety, depression, have a lower quality of life or negative perceptions of being a family caregiver, and this is supported by previous studies [8, 10, 16]. A higher degree of attachment anxiety [21, 22] and the patient having changed behaviour seem also to contribute an extra burden to the family in this context [18]. Even if all of the hypothesized domains were associated with the family members’ sense of support within the closest family, the lack of association with gender, socioeconomic factors, and cultural diversity contrast with those of previous studies [16].

The presence of a child or children, either in the household of the family member or that of the patient, was associated with family members who less frequently had a sense of support within the closest family. These results suggest that children may have a major impact on how family members’ capability of giving support to each other. Several studies have reported that family members who care for an ill relative or friend while also taking care of a child at home report extra challenges, for example due to the splitting of time between caregiving duties and taking care of children, and that they experience a changed relationship with their children [18], and a diminished quality of life [27] in this situation. Members of the more dysfunctional family types (sullen and hostile), with elevated depressive symptoms, are especially likely to perceive poorer relationships with their children [9]. Consequently, it seems important that palliative care practitioners pay specific attention to families having children living at home, irrespective of whether the children are living with the patient or with the patient’s family members, and that this is even more important in families were patients or family members shows symptoms of depression.

Moreover, living alone was associated with greater perceptions of support within the closest family, and being the child of the patient was associated with less perception of such support – both these variables were selected in the final model. The former may have to do with the difficulty of managing multiple household responsibilities in addition to giving care to the patient and/or violations of expectations concerning how much support one’s household should provide [28, 29]. The latter effect may have to do with the special circumstances and burden associated with caring for an aged parent [28, 29].

Another result we would like to draw attention to is that three of the six selected variables in the final model concerned different kinds of support given to the family member or to the patient: *i)* Support to family member from other family members than the closest family; *ii*) Support to patient from other family members, relatives or friends; *iii*) Support from the palliative care unit (in terms of family members’ sense of security in palliative care-Patient Situation subscale). The Patient Situation subscale comprises, for example, questions about the patient having a good situation, despite his or her illness, and the patient receiving health care at a location of the family member’s preference. These findings stress the significance of extending the traditional focus on family member burden in the palliative care context, with a salutogenic perspective that recognizes strengths and resilience-building capacity and coping, e.g. by palliative care staff members supporting family members and patients to identify and accept help from supportive social networks, and by striving to help family members to feel secure with the care given by the palliative care team, for example through adequate management of the patient’s symptoms, and the palliative team involving the family members in the planning of care. Such strategies may facilitate the family’s ability to support each other and reduce the associated psychological morbidity [16, 30].

The findings are interesting not only from a salutogenic perspective, but when applying the Family Systems Theory. As mentioned in the introduction, this theory proposes that the family should be understood as both an interrelated and an interdependent individual component within a hierarchy of subsystems and suprasystems [3, 14, 31]. The functioning of the subsystem “Family member - Closest family” was assessed in this study and also whether either of the two domains “Family member characteristics” and “Patient characteristics” would be associated with the functioning of this subsystem (in terms of family member’s perception of how often the closest family members gave support to each other). The findings supported these assumptions. Secondly, we assessed if the functioning of the two subsystems, “Patient - Other family members” and “Family member – Other more distant family members”, respectively, would be related to the functioning of the “Family member - Closest family”. These hypotheses were also supported by the results. Thirdly, we assessed if the functioning of the subsystem “Family member - Closest family” was related to a suprasystem with the palliative care unit. This hypothesis was likewise supported by the findings. In addition, in the model-building process, six variables from four different domains, indicating two different subsystems and one suprasystem, were selected for predicting family members’ sense of support within the closest family.

Consequently, it seems important – according to the Family Systems Theory framework - that palliative care practitioners regard the family member as one part of several interrelated and interdependent subsystems and larger suprasystems [2, 3, 14], and therefore pay attention to the family member’s as well as the patient’s situation and the support available from the social network, to facilitate the family’s ability to support each other. The results are in line with previous findings that patients and family members constitute a relational system [11] and support further development of interventions in which both patients and family members may have the possibility of participating together [12, 32]. Interventions targeting only the patient or one type of family member, e.g. spouses, may have limited effectiveness and miss the possibility of helping the entire family to support each other and adapt to the challenges.

### Limitations

Some issues should be noted in terms of the validity, reliability and generalisability of the findings. First of all, in the study design, we postulated that examining one element (the family member’s perspective) of a system, which involves several diverse but still related elements (different subsystems as well as the palliative care unit as a suprasystem), leads to a correct description of the whole system. The family member’s perspective of the functioning of the different parts is relevant and of significance per se*.* For example, as a description of the family member perspective within a family system that the practitioner can use to identify family members sensing a low level of support within the closest family, or as a test if the Family System Theory fits the structure of the factors that affect family members’ sense support within the closest family. However, this design has limitations if one generalise one family member’s perspective to reflect the whole of the family. It seems important that future research collect data from several components of the family system and adopt an analytical approach to further explore more complex levels regarding support within the family, e.g. the interdependence and reciprocal nature of support within the family [4].

The study had a cross-sectional design, and hence the results can merely propose causal relations. Additionally, the narrow range of support within the closest family scores limits the generalizability of the results to family members with low scores on this measure. In addition, the analysis did not examine patterns of mediation or interaction, which may have relevance when examining systems of variables. Also, data is collected 8 years back in time and all data is collected in Sweden. During that time period there has not been any large changes regarding the organisation of the palliative care, possibly the length of stay in hospital has become even shorter since the data were collected. This makes it more important to recognise patients and family members needs in the palliative home care context.

## Conclusion

This paper identified 19 variables associated with the family members’ sense of support within the closest family. A model was developed, including six variables, that predicted the family members’ sense of support within the closest family; family member perceiving support from other more distant family members; feeling secure with the provided palliative home care; possibility of respite if family member needed a break; family member living alone; being a child of the patient; perceiving that the patient was supported by other family members. Our findings support the clinical relevance of applying the Family Systems Theory in the palliative care context since it could help palliative care practitioners select proper supportive family interventions, and to evaluate their effectiveness.

## Data Availability

The datasets generated and analyzed during the current study are not publicly available because of research participants’ privacy.

## Abbreviations

AIC: The Akaike Information Criterion
SEC-R: Patient Situation subscale
